# Enhanced Bacterial Cuproptosis‐Like Death via Reversal of Hypoxia Microenvironment for Biofilm Infection Treatment

**DOI:** 10.1002/advs.202308850

**Published:** 2024-03-13

**Authors:** Zhiyuan Luo, Renjie Lu, Tingwang Shi, Zesong Ruan, Wenbo Wang, Zhao Guo, Zeming Zhan, Yihong Ma, Xiaofeng Lian, Cheng Ding, Yunfeng Chen

**Affiliations:** ^1^ Department of Orthopedic Surgery Shanghai Institute of Microsurgery on Extremities Shanghai Sixth People's Hospital Affiliated to Shanghai Jiao Tong University School of Medicine 600 Yishan Road Shanghai 200233 China

**Keywords:** biofilm, enhanced cuproptosis‐like death, hypoxia microenvironment, immunomodulation, respiratory metabolism

## Abstract

A recently emerging cell death pathway, known as copper‐induced cell death, has demonstrated significant potential for treating infections. Existing research suggests that cells utilizing aerobic respiration, as opposed to those reliant on glycolysis, exhibit greater sensitivity to copper‐induced death. Herein, a MnO_2_‐loaded copper metal–organic frameworks platform is developed denoted as MCM, to enhance bacterial cuproptosis‐like death via the remodeling of bacterial respiratory metabolism. The reversal of hypoxic microenvironments induced a cascade of responses, encompassing the reactivation of suppressed immune responses and the promotion of osteogenesis and angiogenesis. Initially, MCM catalyzed O_2_ production, alleviating hypoxia within the biofilm and inducing a transition in bacterial respiration mode from glycolysis to aerobic respiration. Subsequently, the sensitized bacteria, characterized by enhanced tricarboxylic acid cycle activity, underwent cuproptosis‐like death owing to increased copper concentrations and aggregated intracellular dihydrolipoamide S‐acetyltransferase (DLAT). The disruption of hypoxia also stimulated suppressed dendritic cells and macrophages, thereby strengthening their antimicrobial activity through chemotaxis and phagocytosis. Moreover, the nutritional effects of copper elements, coupled with hypoxia alleviation, synergistically facilitated the regeneration of bones and blood vessels. Overall, reshaping the infection microenvironment to enhance cuproptosis‐like cell death presents a promising avenue for eradicating biofilms.

## Graphical abstract

Herein, an enhanced strategy is proposed for bacterial cuproptosis‐like cell death via the remodeling of bacterial respiratory metabolism. The reversal of hypoxic microenvironments induced a cascade of responses, encompassing the reactivation of suppressed immune responses and the promotion of osteogenesis and angiogenesis. The comprehensive amelioration of infections demonstrates significant potential for clinical applications.

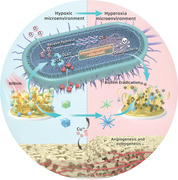

## 1 Introduction

One of the most prevalent and severe complications in orthopedic surgery is the occurrence of implant‐associated infections (IAIs), wherein bacteria colonize and form biofilms on the surface of the implant.^[^ ^1^ ^]^ In established biofilms, pathogenic bacteria strongly adhere to the surface of the biological substrate.^[^ ^2^ ^]^ The infective microenvironment within the biofilm is characterized by acidic and hypoxic conditions, which promote enhanced bacterial pathogenicity and survival.^[^ ^3^ ^]^ Conventional antimicrobial therapies often concentrate on eradicating pathogenic bacteria while overlooking the regulation of the infection microenvironment, leading to challenges such as antibiotic resistance and incomplete antimicrobial efficacy.^[^ ^4^ ^]^ Thus, simultaneously targeting both bacteria and the infection microenvironment of the biofilm could potentially offer a novel approach to biofilm eradication.

Copper is an ancient and classic metallic element that finds extensive application in the field of antimicrobial research.^[^ ^5^ ^]^ Copper accumulation within biological membranes has been recently found to trigger copper‐induced cell death through interaction with components in the tricarboxylic acid (TCA) cycle.^[^ ^6^ ^]^ Copper‐induced cell death has demonstrated significant potential for cancer treatment.^[^ ^7^ ^]^ Notably, Tsvetkov et al. indicated that the targets of copper‐induced death are evolutionarily conserved from humans to bacteria, and Mei et al. demonstrated the existence of bacterial cuproptosis‐like death.^[^ ^8^ ^]^ However, the efficacy of copper‐induced death is significantly restricted by the hypoxic microenvironment within the biofilm.^[^ ^9^ ^]^ Previous studies have corroborated the correlation between copper‐induced cell death and cellular respiration modes, wherein cells reliant on aerobic respiration demonstrate a sensitivity to copper‐induced death that is a thousand times higher than that of glycolysis‐dependent cells.^[^ ^10^ ^]^ Therefore, addressing the obstacles posed by this hypoxic environment to enhance copper‐induced cell death presents a challenging and inevitable undertaking.

The hypoxic environment within biofilms is also associated with immune cell suppression.^[^ ^11^ ^]^ Research has revealed that immune cells require O_2_ to generate immunostimulatory NO and cytotoxic substances, which are crucial for effectively eliminating bacteria.^[^ ^12^ ^]^ Additionally, hypoxic conditions can hinder the phagocytic activity of monocytes, thereby suppressing their ability to engulf bacteria.^[^ ^13^ ^]^ Consequently, disrupting the hypoxic microenvironment within biofilms could potentially lead to the comprehensive activation of the immune system. Moreover, copper elements, which serve as crucial immunomodulators, actively facilitate the differentiation of immune cells, thereby stimulating immune responses and expediting the clearance of infections.^[^ ^14^ ^]^

The growth of local bone tissue and neovascularization following bacterial clearance is crucial for treating infections.^[^ ^15^ ^]^ Moreover, the importance of local oxygenation in wound healing is well‐established.^[^ ^16^ ^]^ Alleviating the hypoxic environment supports the metabolism and regeneration of local cells. Extensive research has been conducted on the facilitative role of copper elements in osteogenesis and angiogenesis.^[^ ^17^ ^]^ Zhang et al., in a study employing a combination of copper‐based nanomaterials and photothermal therapy, observed a marked acceleration in local bone growth following treatment.^[^ ^18^ ^]^

**Scheme 1:**
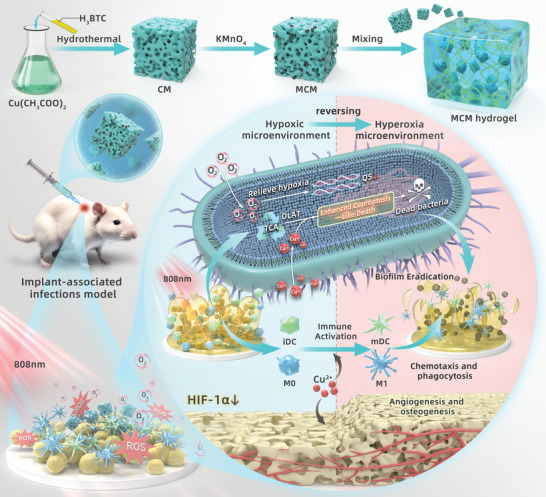
Schematic of the preparation of MnO_2_ loaded copper metal–organic frameworks (MCM) and the multiple synergistic antibacterial strategies based on reshaping the infection microenvironment to enhance bacterial cuproptosis‐like death, reawakening suppressed immune responses, and promoting osteogenesis and angiogenesis.

In this study, we propose an enhanced strategy for bacterial cuproptosis‐like cell death by reversing hypoxic microenvironments (**Scheme** **1**). We integrated multiple therapeutic functions, including reactivation of suppressed immune responses and promotion of osteogenesis and angiogenesis, into a single nanoplatform, MnO_2_‐loaded copper metal–organic frameworks (MCM). The loaded MnO_2_ catalyzed O_2_ production through the reaction of H_2_O_2_ within the biofilm. With the supplementation of O_2_ and copper, bacterial respiration transitioned from glycolysis to aerobic respiration, accompanied by an increase in intracellular copper concentration and dihydrolipoamide S‐acetyltransferase (DLAT) oligomerization, ultimately resulting in enhanced cuproptosis‐like cell death. Both in vitro and in vivo experiments confirmed the substantial efficacy of combining MCM and photothermal therapy for eradicating biofilms. The reactivation of dendritic cells (DCs) and macrophages following O_2_ supply was also confirmed, and the alleviation of hypoxia and copper nutrition facilitated osteogenesis and angiogenesis after infection clearance. Notably, our strategy of remodeling bacterial metabolism to enhance cuproptosis‐like cell death may serve as a new paradigm and direction for augmenting existing therapeutic approaches. The comprehensive amelioration of infections demonstrates significant potential for clinical applications.

## 2 Results and Discussion

### 2.1 Synthesis and Characterization of MCM

**Figure 1:**
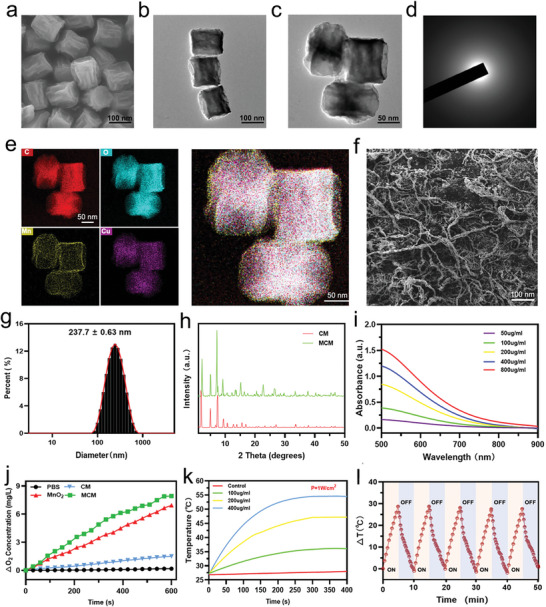
Characterization of CM and MCM. a) SEM images of CM. Scale bar, 100 nm. b,c) TEM images of MCM. Scale bar, 100 nm, 50 nm. d) SEAD of MCM. e) Elemental mapping images of MCM. Scale bar, 50 nm. f) SEM images of ECMH mixed with MCM. Scale bar, 100 nm. g) Particle size distribution of MCM. h) XRD of CM and MCM. i) UV–vis spectra of MCM at varied concentration (50, 100, 200, 400, and 800 µg mL^−1^). j) O_2_ production in H_2_O_2_ solution treated with phosphate buffered saline (PBS), MnO_2_, CM, and MCM. k) Temperature elevation curve of MCM solution at varied concentration (0, 100, 200, and 400 µg mL^−1^) upon NIR laser irradiation (P = 1 W cm^−2^). l) Heating and cooling curves of MCM (400 µg mL^−1^) after five cycles of 808 nm laser irradiation (P = 1 W cm^−2^). Data are presented as they are.

To construct a synergistic therapeutic nanoplatform, we synthesized MCM following established procedures.^[^ ^19^ ^]^ Core nanoparticles (NPs) of copper metal–organic frameworks (CM) were synthesized using a hydrothermal method. Prepared copper acetate monohydrate was mixed with 1,3,5‐Benzenetricarboxylic acid (H_3_BTC) and stirred for 12 h to obtain CM. Scanning electron microscopy (SEM) revealed the polyhedral shape of CM NPs, characterized by a uniform size (mean particle size 214.3 ± 4.11 nm) (**Figure** **1**a; Figure S1, Supporting Information). Bare CM NPs exhibited a positively charged surface (zeta potential 30.0 ± 0.9 eV) (Figure S2, Supporting Information). Subsequently, utilizing the CM shell as a substrate, we applied an MnO_2_ coating to the NP surface through the reduction of KMnO_4_, facilitated by polyallylamine hydrochloride (PAH), resulting in the generation of MCM. The prepared NPs were characterized using transmission electron microscopy (TEM) and dynamic light scattering (DLS). TEM revealed the polyhedral shape of MCM, whereas DLS showed that the mean particle size of MCM was 237.7 ± 0.63 nm (Figure 1b–g). The observed increase in diameter, compared to electron microscopy observations, may be attributed to the absorption of water molecules on the surfaces of CM and MCM.^[^ ^20^ ^]^ The surface of MCM was negatively charged (zeta potential −10.0 ± 0.7 eV) (Figure S2, Supporting Information), indicating the successful modification of MnO_2_. The amorphous structure of MCM was determined through the analysis of selected area electron diffraction (SAED) patterns (Figure 1d). Subsequently, MCM was further characterized, and element mapping results showed that Cu, Mn, O, and C were uniformly distributed on MCM, with Mn originating from MnO_2_, further supporting the successful modification of MnO_2_ (Figure 1e). For the practical in vivo application of MCM, we employed an extracellular matrix hydrogel (ECMH), a commonly used hydrogel with excellent biocompatibility and biodegradability, as a carrier for MCM. SEM was used to characterize the microstructure of the ECMH (Figure 1f). The hydrogel exhibited increased viscosity and possessed a black color after mixing with MCM (Figure S3, Supporting Information).

For further characterization, the X‐ray powder diffraction (XRD) analysis implied the successful modification of MnO_2_ on the metal–organic framework (Figure 1h). The MCM solution exhibited a wide optical absorption spectrum ranging from 550 to 850 nm (Figure 1i). Moreover, sustained O_2_ generation reversed the hypoxic microenvironment and triggered synergistic effects (Figure 1j). The catalytic capabilities of CM and MnO_2_ in the generation of oxygen were also assessed, and no significant enhancement effect was observed upon combining CM with MnO_2_. Regarding photothermal property, the temperature of the MCM solution (100 µg mL^−1^) increased rapidly under near‐infrared (NIR) laser irradiation (808 nm, 1 W cm^−2^). At NPs concentrations of 200 and 400 µg mL^−1^, temperature increases of 20 and 28 °C were observed, respectively (Figure 1k; Figure S4, Supporting Information). Upon evaluating photothermal stability, a rapid temperature increase was observed when the MCM aqueous solutions were exposed to an 808 nm laser. Minimal alterations in photothermal conversion efficiency for five heating and cooling cycles were observed, indicating the remarkable photostability of MCM (Figure 1l). Furthermore, utilizing the classical heat exchange method, we calculated the photothermal conversion efficiency of MCM to be 39.15%, demonstrating its good photothermal performance (Figure S5, Supporting Information). To assess the biocompatibility of MCM, a cell counting kit‐8 (CCK‐8) assay was conducted, which revealed no significant cytotoxicity until the concentration reached 800 µg mL^‐1^ (Figure S6, Supporting Information). Consequently, considering the combined photothermal properties and biocompatibility of MCM, a concentration of 400 µg mL^−1^ was deemed optimal for therapeutic applications.

### 2.2 Anti‐Biofilm Activity of MCM + NIR In Vitro

MCM, in combination with photothermal treatment, efficiently eradicated biofilms through enhanced cuproptosis‐like cell death. To assess the anti‐biofilm effects of MCM, six experimental groups were established: control, CM, MCM, NIR, CM + NIR, and MCM + NIR. *Staphylococcus aureus (S. aureus)* was selected as the representative IAI bacteria. Before NIR irradiation, biofilms were pretreated with CM or MCM for 8 h. For the NIR groups, laser irradiation was applied for 10 min, and monitoring was conducted using an infrared imaging device.

**Figure 2:**
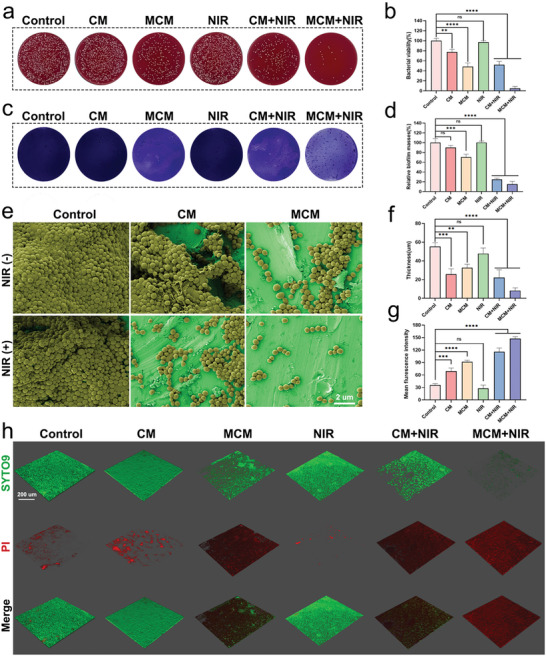
Antibiofilm properties of MCM. a) Representative photographs depicting bacterial colonies within *S. aureus* biofilms using SPM. b) Quantitative evaluation of bacterial viability via SPM. c) Digital images of the *S. aureus* biofilms stained with crystal violet. d) Biomass of *S. aureus* biofilm after different treatments. e) SEM images of *S. aureus* biofilms. Scale bar, 2 µm. f) Quantification analysis of 3D‐reconstructed biofilm thicknesses. g) Mean fluorescence intensity of red stained biofilms (dead bacteria). h) 3D reconstruction images presenting *S. aureus* biofilm stained with SYTO 9/ PI. Scale bar, 200 µm. Data are presented as mean ± S.D, n =  3, ^**^ *p* < 0.01, ^***^ *p* < 0.001, and ^****^ *p* < 0.0001.

The viability of bacteria in the biofilm after various treatments was assessed using the spread plate method (SPM) (**Figure** **2**a). Quantitative analysis indicated that the MCM + NIR treatment effectively eradicated most bacteria within the biofilm (Figure 2b). Crystal violet staining revealed that the control group effectively maintained biofilm integrity, whereas the MCM and MCM + NIR groups exhibited a significant reduction in biofilm biomass (Figure 2c,d). The SEM results were consistent with the above findings, showing a significant reduction in the number of bacteria on the surface of titanium sheets following treatment with MCM or MCM + NIR, demonstrating the excellent anti‐biofilm capabilities of MCM (Figure 2e). Confocal laser scanning microscopy (CLSM) was conducted to qualitatively assess the 3D disruption of the biofilm. In the control group, green fluorescence representing viable bacteria was strong and densely distributed, whereas red fluorescence representing dead bacteria was sparse and fragmented (Figure 2h). A rapid decrease in green fluorescence and a significant increase in red fluorescence were observed in the MCM and MCM + NIR groups, indicating a substantial loss of bacterial vitality. Quantitative analysis revealed that the biofilm thickness in the MCM and MCM + NIR groups decreased to 32.56 and 8.16 µm, respectively, confirming the inhibitory effect of MCM on biofilm stacking (Figure 2f,g). The differences in bacterial survival rates between the CM + NIR and MCM + NIR groups may be attributed to the presence of supplemental O_2_. The specific underlying mechanisms will be further investigated in subsequent studies. To gain a comprehensive understanding of MCM's antimicrobial properties, we selected Escherichia coli (*E. coli)* as the representative of Gram‐negative bacteria. As illustrated in Figures S7–S9 (Supporting Information), the experimental results demonstrate significant antibacterial effects of MCM against *E. coli*.

### 2.3 MCM Remodeled Bacterial Respiratory Metabolism

**Figure 3:**
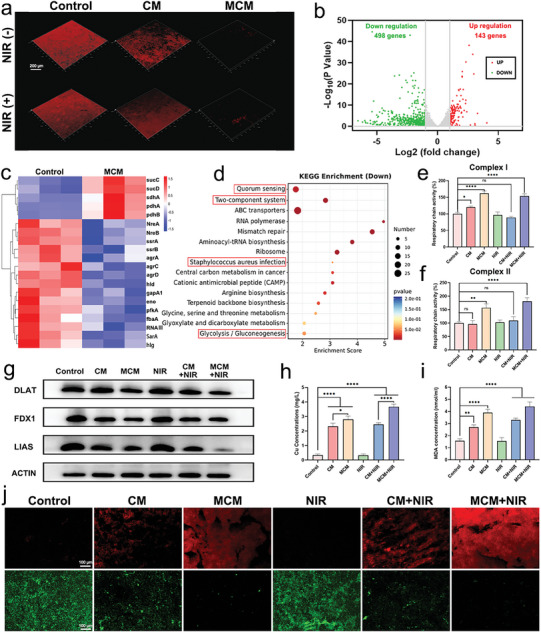
MCM remodeled bacterial respiratory metabolism and induced enhanced cuproptosis‐like death. a) Immunofluorescence images of *S. aureus* biofilm staining with hypoxyprobe in different groups. Scale bar, 200 µm. b) Volcano plots of differentially expressed genes (green: upregulated genes; red: downregulated genes). c) Heat map of differentially expressed genes among *S. aureus* biofilm treated with PBS or MCM. d) KEGG enrichment of downregulated pathways. e–f) Activity of respiratory chain complex I and II in *S. aureus* biofilm with different treatments. g) Western blotting results of bacterial proteins involved with cuproptosis‐like death. h) The intracellular copper concentration quantified using ICP‐OES. i) MDA content within *S. aureus* biofilms across different experimental groups. j) *S. aureus* biofilm stained with lipid peroxidation fluorescent probe (C11 BODIPY) and SytoX. Scale bar, 100 µm. Data are presented as mean ± S.D, n =  3, ^*^ *p* < 0.05, ^**^ *p* < 0.01, and ^****^ *p* < 0.0001.

Bacterial infections create a microenvironment characterized by hypoxia,^[^ ^21^ ^]^ with O_2_ concentration within the biofilm decreasing significantly with increasing depth.^[^ ^22^ ^]^ Consequently, bacteria located at the base of the biofilm rely primarily on glycolysis as their main mode of respiration.^[^ ^23^ ^]^ MCM targeted and ameliorated the hypoxic conditions within the biofilm and remodeled bacterial respiratory metabolism through O_2_ supplementation, thereby inducing enhanced copper‐induced cell death. A hypoxia probe was employed to investigate the oxygenation status within the biofilm. Compared to the control group, the MCM and MCM + NIR groups exhibited a significant reduction in hypoxic signals, indicating a pronounced alleviation of hypoxic conditions (**Figure** **3**a). To clarify the mechanism underlying the MCM‐induced regulation of bacterial metabolism, we examined the gene expression of *S. aureus* using RNA sequencing. Significant variations in gene expression were observed following MCM treatment. Compared to the control group, the MCM group exhibited 498 downregulated and 143 upregulated genes (Figure 3b). Heatmap analysis revealed that MCM treatment led to a substantial upregulation of genes associated with the bacterial TCA cycle, including *sucC*, *sucD*, and *sdhA* (Figure 3c). Conversely, glycolysis‐related genes, such as *gapA1*, *eno*, and *pfkA* were significantly downregulated. Kyoto Encyclopedia of Genes and Genomes (KEGG) analysis revealed a significant decline in the glycolytic pathway (Figure 3d). Furthermore, pathways associated with bacterial pathogenicity and biofilm formation, including quorum sensing and *S. aureus* infection, were also included in the findings. The activities of complexes I and II in the electron transport chain (ETC) were assessed to investigate the effect of O_2_ concentration on bacterial respiratory metabolism (Figure 3e,f). The increased activity of complexes I and II indicated a substantial alteration in the bacterial respiratory mode. In conclusion, RNA sequencing demonstrated that our nanomedicine induced a transition in bacterial respiration, shifting from glycolysis to aerobic respiration characterized by an enhanced TCA cycle.

### 2.4 MCM Induced Enhanced Cuproptosis‐Like Cell Death

Previous studies have established a connection between copper‐induced cell death and cellular respiration patterns.^[^ ^10^ ^]^ Specifically, cells reliant on aerobic respiration exhibit a sensitivity to copper‐induced cell death that is a thousand times greater than that of glycolysis‐dependent cells. To demonstrate the augmented bacterial cuproptosis‐like death, western blotting analysis was conducted to assess the expression levels of marker proteins within the associated signaling pathways. During cuproptosis‐like cell death, DLAT bound to Cu^+^ to form insoluble oligomeric DLAT. Figure 3g showed the decreased expression of DLAT protein and the other two marker proteins, LIAS and FDX1. The oligomerization of DLAT was also observed by western blotting (Figure S10, Supporting Information). Copper content within the biofilm was detected using inductively coupled plasma optical emission spectrometry (ICP‐OES), which indicated a significant increase in copper concentration in the MCM and MCM + NIR groups (Figure 3h). The accumulation of malondialdehyde (MDA), a metabolic product associated with lipid peroxides, was demonstrated using a reagent kit after MCM treatment (Figure 3i). Finally, the biofilm was stained with a C11 BODIPY 581/591 fluorescent probe and SYTOX to observe internal lipid peroxidation and bacterial viability, respectively. Lipid peroxidation was significantly enhanced after MCM treatment, and SYTOX staining indicated the disruption of the bacterial biofilm (Figure 3j). Notably, differences between the CM and MCM groups were observed, demonstrating the effectiveness of O_2_ supplementation in enhancing copper‐induced cell death. Overall, MCM enhanced cuproptosis‐like cell death by alleviating hypoxia and supplying copper.

### 2.5 MCM Reactivated Suppressed Immune Responses In Vitro

**Figure 4:**
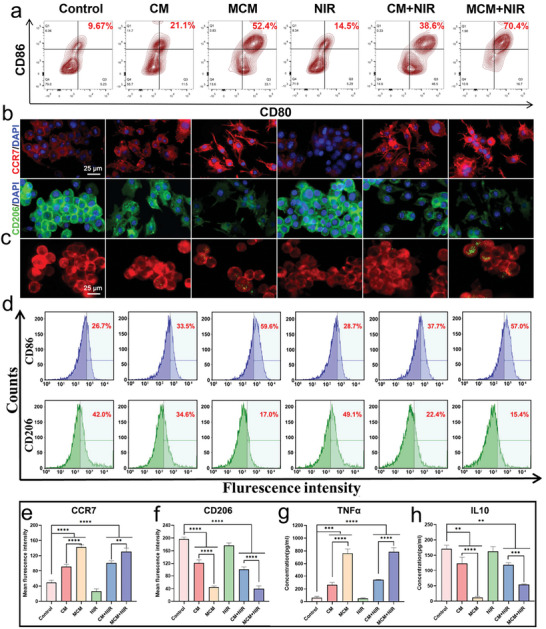
MCM reinvigorated macrophages and dendritic cells. a) Typical scatter plots of dendritic cell after treatment. b) Immunofluorescent staining of RAW264.7 macrophages. CCR7 (red, M1), CD206 (green, M2), and DAPI (blue). Scale bar, 25 µm. c) Typical visuals displaying the phagocytosis of bacteria by macrophages. Red fluorescence corresponds to macrophages, green fluorescence corresponds to *S. aureus*. Scale bar, 25 µm. d) Flow cytometric analysis of CD86 (M1) and CD206 (M2) expression in macrophages. e,f) Quantitative analysis of immunofluorescent results. g,h) ELISA results of proinflammatory and anti‐inflammatory cytokines (TNFα and IL‐10). Data are presented as mean ± S.D, n =  3, ^**^ *p* < 0.01, ^***^ *p* < 0.001, and ^****^ *p* < 0.0001.

Previous studies have substantiated the fundamental role and significance of copper ions in immunostimulatory effects.^[^ ^24^ ^]^ Hypoxic conditions within the biofilm have also been demonstrated to impede the bactericidal ability of the immune cells.^[^ ^11^ ^]^ The immunosuppressive effects of biofilm infection microenvironments promote the retention of bacteria within the host.^[^ ^3^ ^]^ Therefore, disrupting this immunosuppressive state is crucial to prevent the persistence of chronic infections. Given that DCs are crucial antigen‐presenting cells that play fundamental roles in the immune system, we subjected them to MCM treatment and assessed their capacity to promote cell differentiation.^[^ ^25^ ^]^ Flow cytometry was used to assess DC maturation via an analysis of the expression levels of the co‐stimulatory molecules CD80 and CD86 (**Figure** **4**a). The results indicated that the percentage of mature DCs (CD80^+^CD86^+^) following incubation with MCM was significantly higher (70.4%) than that of the control group. Macrophages have recently emerged as significant targets for immunotherapy owing to their controllable phenotypic transitions.^[^ ^26^ ^]^ The CLSM images demonstrated that MCM treatment resulted in an increase in red fluorescence, indicating enhanced CCR7 expression (an M1 marker), and a decrease in green fluorescence, indicating reduced CD206 expression (an M2 marker) (Figure 4b). Quantitative analysis of the fluorescence intensity further corroborated these findings (Figure 4e,f). Subsequently, we investigated changes in the ability of macrophages to phagocytose bacteria. Numerous engulfed bacteria were observed within cells in the MCM and MCM + NIR groups (Figure 4c). The flow cytometry analysis results corroborate earlier experimental findings (Figure 4d). The proportion of M1 cells increased to 59.6% and 57% in the MCM and MCM + NIR groups, respectively. Moreover, enzyme‐linked immunosorbent assay (ELISA) indicated an upregulation of the pro‐inflammatory cytokine TNFα and a downregulation of the anti‐inflammatory cytokine IL10 (Figure 4g,h). Overall, MCM promoted DC maturation and induced macrophage M1 differentiation, thereby reactivating the suppressed immune system. The enhanced phagocytic activity and chemotactic responses facilitated biofilm removal.

### 2.6 MCM Promoted Osteogenesis and Angiogenesis In Vitro

**Figure 5:**
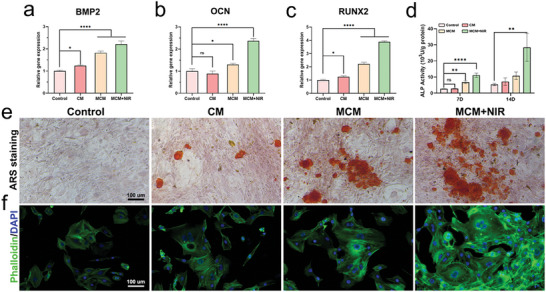
Osteogenetic and angiogenesis effects of MCM. a–c) Expression of osteogenic genes (*BMP2*, *OCN*, and *RUNX2*) accessed by RT‐qPCR assay. d) ALP activity measured after culturing for 7 and 14 days. e) Cellular mineralization visualized through ARS staining. Scale bar, 100 µm. f) Fluorescence images of phalloidin and DAPI co‐stained images of BMSCs. Scale bar, 100 µm. Data are presented as mean ± S.D, n =  3, ^*^ *p* < 0.05, ^**^ *p* < 0.01, and ^****^ *p* < 0.0001.

We assessed the potential of MCM to promote osteogenesis and angiogenesis. Real‐time quantitative reverse transcription polymerase chain reaction (RT‐qPCR) analysis was conducted on a series of genes associated with osteogenesis in bone marrow‐derived mesenchymal stem cells (BMSCs) (**Figure** **5**a–c). Compared to the control group, the MCM group showed significantly increased expression of *BMP2*, *OCN*, and *RUNX2*.^[^ ^27^ ^]^ Furthermore, ALP activity was investigated to determine the osteogenic differentiation capacity of the cells under different treatments. The results were consistent with those of the PCR analysis (Figure 5d). Extracellular osteogenic mineralization, as reflected by alizarin red staining (ARS), was enhanced in both the MCM and MCM + NIR groups (Figure 5e). Given that the cell size and proliferative differentiation of BMSCs reflect their osteogenic capability, we performed 4′,6‐diamidino‐2‐phenylindole (DAPI) and phalloidin staining to assess the morphology of BMSCs. Noticeably broader BMSCs were observed in the MCM group (Figure 5f). Hypoxia alleviation and the nutritional effects of copper can also promote angiogenesis.^[^ ^28^ ^]^ A tube formation assay was conducted, and more junctions and branches, which are classic indicators of vascular formation, were observed in the MCM group (Figure S11, Supporting Information).

### 2.7 Anti‐Infection and Immunomodulatory Effects of MCM In Vivo

**Figure 6:**
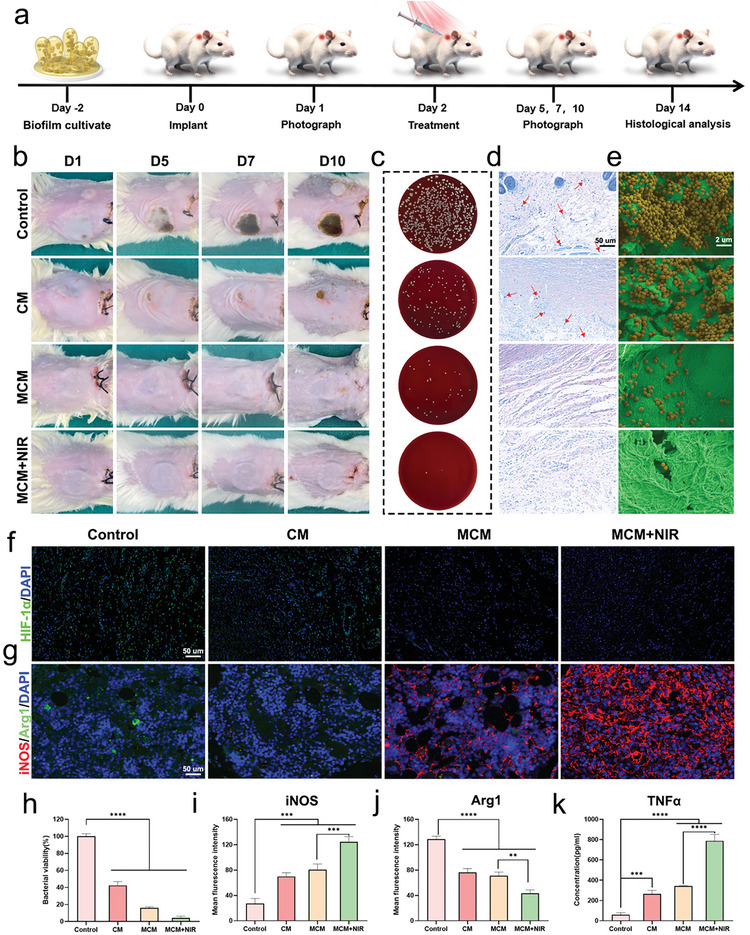
Antimicrobial activity and immunomodulatory effects of MCM in vivo. a) Schematic design of constructing IAI model and in vivo treatment procedures. b) Digital photographs of IAI models on days 1, 5, 7, 10, and 14. c) Bacterial colonies of cultures from wound tissues. d) Giemsa staining images of wound tissues. The red arrows pinpoint the stained bacteria. Scale bar, 50 µm. e) SEM images of *S. aureus* biofilms in vivo. Scale bar, 2 µm. f) Immunofluorescent staining of HIF‐1α with different treatments (green fluorescence indicates HIF‐1α; blue fluorescence indicates cell nucleus). Scale bar, 50 µm. g) Immunofluorescent staining of RAW264.7 cells. Red fluorescence represents iNOS (M1 macrophage marker), and green fluorescence represents Arg‐1 (M2 macrophage marker). Scale bar, 50 µm. h) Quantitative analysis of relative newborn skin and bacterial viability. i,j) Quantitative analysis of immunofluorescent results. k) ELISA results of TNFα in vivo. Data are presented as mean ± S.D, n =  6, ^**^ *p* < 0.01, ^***^ *p* < 0.001, and ^****^ *p* < 0.0001.

Considering the multifunctional therapeutic capabilities of MCM in vitro, we investigated its antimicrobial and immunomodulatory capacities using an IAI model in vivo.^[^ ^29^ ^]^ Four groups were used for the in vivo experiments: control, CM, MCM, and MCM + NIR. The experimental procedure is illustrated in **Figure** **6**a. A digital camera was used to capture images on days 1, 5, 7, 10, and 14 to record the infection and skin conditions. Pronounced purulent exudates were observed in the control and CM groups, indicating severe inflammation (Figure 6b). Subsequently, SPM and Giemsa staining were used to validate the in vivo antimicrobial efficacy of MCM (Figure 6c,d). Compared to the control group, bacterial viability in the MCM and MCM + NIR groups decreased to 16% and 4.2%, respectively (Figure 6h). The electron microscopy results were consistent with these findings, further confirming the antimicrobial efficacy of MCM (Figure 6e). Furthermore, hematological analysis was conducted on peripheral blood samples obtained from mice (Figure S12, Supporting Information). The experimental group exhibited a notable decrease in white blood cell count, indicating infection clearance and reduced inflammation. No significant differences were observed among the groups in red blood cells, hemoglobin, and platelet levels. As previously described, the various therapeutic effects of MCM stem from its ability to reverse the hypoxic microenvironment. Therefore, the potential in vivo hypoxia mitigation effect of MCM was assessed through HIF‐1α immunofluorescence staining. The gradual decrease in fluorescence intensity indicated the alleviation of tissue hypoxia (Figure 6f).

Additionally, we performed immunofluorescence assessments using the M1 marker iNOS and the M2 marker ARG1 to offer supplementary proof of the in vivo immunomodulatory effects of MCM (Figure 6g). Notably, a substantial infiltration of M1 macrophages was observed in both the MCM and MCM + NIR groups. Quantitative analysis of the fluorescence intensity is presented in Figure 6i,j. ELISA was used to analyze the expression levels of cytokines, and the results were consistent with the immunofluorescence results (Figure 6k; Figure S13, Supporting Information). These results demonstrate that MCM effectively mitigated infection by alleviating hypoxia and immunostimulation in vivo. The biodistribution of MCM in vivo was assessed by inductively coupled plasma‐mass spectrometry (ICP‐MS) and no significant differences in the distribution of organs among the various groups were observed (Figure S14, Supporting Information).

### 2.8 MCM Promoted Osteogenic and Angiogenesis In Vivo

**Figure 7:**
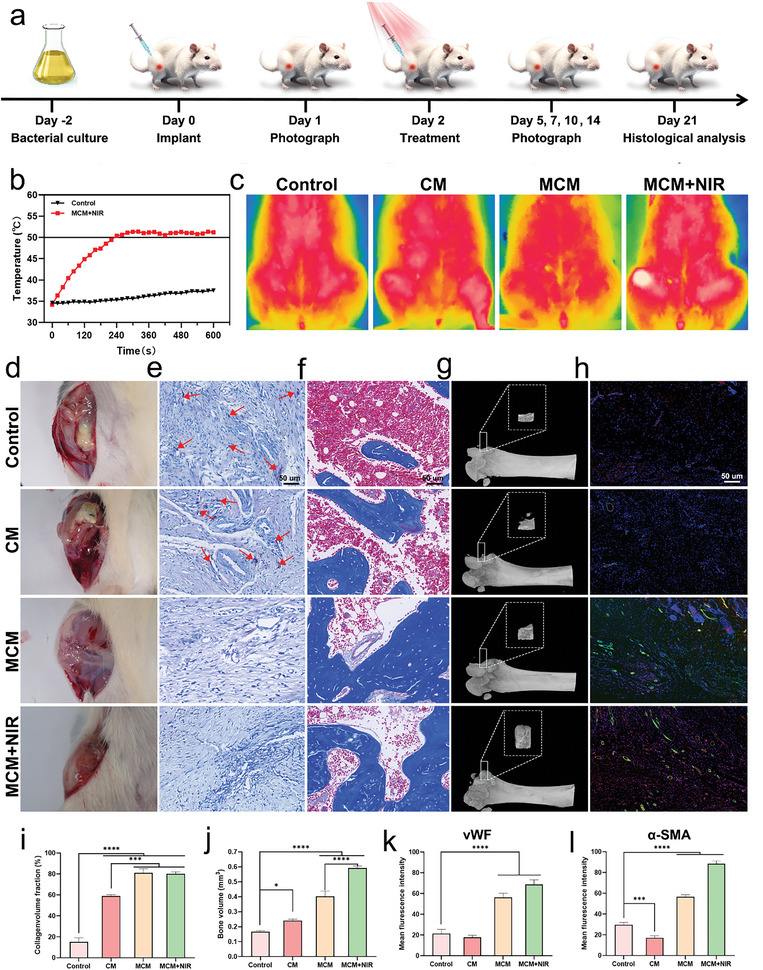
Osteogenic and Angiogenesis properties of MCM in vivo. a) Schematic design of constructing bacterial infected bone defect model and in vivo treatment procedures. b) The temperature‐increase curves of the control and MCM + NIR groups and c) the thermal images. d) Digital photos of the wound. e) Giemsa staining images of wound tissues. The red arrows pinpoint the stained bacteria. Scale bar, 50 µm. f) Masson's staining images of collagen fibers. Scale bar, 50 µm. g) 3D reconstructed micro‐CT images. h) Immunofluorescent staining of von Willibrand factor (vWF) shown in red and α‐SMA shown in green revealing vascular regeneration. Scale bar, 50 µm. i,j) Quantitative analyses of masson staining tests and bone volume. k,l) Quantitative analysis of immunofluorescent results. Data are presented as mean ± S.D, n =  6, ^*^ *p* < 0.05, ^***^ *p* < 0.001, and ^****^ *p* < 0.0001.

A bacterial‐infected bone defect model was used to assess the osteogenic and angiogenic capabilities of MCM.^[^ ^30^ ^]^ A circular defect was created on the femoral condyle, followed by the injection of 10^7^ colony‐forming units of *S. aureus* to induce bone infection. The procedural steps and time points of the animal experiments are shown in **Figure** **7**a. Bacteria were cultured on a shaker for 48 h. On days 0 and 1, respectively, the rat bone infection model was established, and gross photographs were taken to document the general condition of the rats. On day 2, the NIR groups were exposed to laser irradiation, and an infrared thermometer was used to monitor and ensure therapeutic temperature. The temperature in the MCM + NIR group rapidly increased to over 50 °C and was maintained within this range for 5 min, whereas the temperature in other groups showed no significant changes (Figure 7b). On day 21, the rats were euthanized, and a series of experiments, including gross photography, immunofluorescence, and micro‐CT, were conducted to assess the therapeutic efficacy of MCM. The control and CM groups exhibited pronounced swelling and ulceration, whereas MCM and MCM + NIR showed significantly milder signs of infection and inflammation (Figure 7d). Giemsa staining was used to assess the residual bacterial load within the tissues. More bacteria (indicated by red arrows) were observed in the control group (Figure 7e).

The osteogenic and angiogenic capabilities of MCM were demonstrated in vitro, prompting further investigation in vivo. Local inflammation and new bone formation were assessed using Masson's and hematoxylin and eosin (H&E) staining (Figure 7f; Figure S15, Supporting Information). Quantitative analysis of collagen deposition revealed significant regeneration of the femoral condyle following MCM treatment (Figure 7i). Moreover, H&E staining revealed a substantial infiltration of inflammatory cells in the control group, whereas a considerable decrease was observed in the MCM group. 3D reconstruction images from micro‐CT revealed that MCM, combined with photothermal therapy, induced new bone formation at the bone defect site, whereas minimal new bone formation was observed in the control group (Figure 7g). Bone volume and surface area were quantified, indicating a significant increase in the MCM group (Figure 7j; Figure S16, Supporting Information). Immunofluorescence staining was performed for two vascular formation‐associated markers, vWF and α‐SMA, to investigate the angiogenic activities of MCM. We selected the soft tissues surrounding bone defects as observational focus. The red and green fluorescent signals, indicative of vascular regeneration, were significantly enhanced following MCM treatment (Figure 7h–l). These results validated the in vivo capacity of MCM to promote the generation of bone and blood vessels. Finally, we performed H&E staining of the major rat organs, including the heart, liver, spleen, lungs, and kidneys (Figure S17, Supporting Information). The results revealed no significant damage, demonstrating the superior biocompatibility of MCM.

## 3 Conclusion

In summary, based on the reversal of hypoxic microenvironments, we constructed a multifunctional nano‐platform for enhanced cuproptosis‐like death and a cascade of therapeutic effects including immunomodulation and tissue regeneration. CCK8 assays and H&E staining of major organs confirmed the high biocompatibility of the MCM. Interestingly, in addition to effects on respiratory metabolism, RNA sequencing revealed the downregulation of pathways associated with biofilm formation, such as quorum sensing, indicating the comprehensive impact of O_2_ supply on bacterial metabolism. Copper‐induced cell death has recently shown significant potential for eradicating bacteria and eliminating tumor cells. We hope that our strategy of remodeling of respiratory metabolism to enhance cuproptosis‐like death will offer a new perspective in the fields of infection and cancer therapy.

## 4 Experimental Section

### Synthesis of MCM

MCM was constructed via hydrothermal synthesis as reported previously.^[^ ^19^ ^]^ First, CM core nanoparticles were synthesized through a hydrothermal process. Copper acetate monohydrate was combined with H_3_BTC and stirred for 12 h, resulting in the formation of CM. Afterward, utilizing the CM shell as a foundation, a MnO_2_ coating was applied to the nanoparticle surface through the reduction of KMnO_4_, facilitated by PAH, resulting in the creation of MCM.

### Oxygen Generation

H_2_O_2_ solutions (25 × 10^−5^ mol L^−1^) were incubated with PBS, MnO_2_, CM, and MCM. The real‐time detection of oxygen production was performed utilizing a dissolved oxygen gauge (HANNA instruments, Korea).

### Photothermal Properties of MCM

Various concentrations of MCM (0, 100, 200, 400 µg mL^−1^) were exposed to laser irradiation with a power density of 1 W cm^−2^ for 5 min, and an infrared thermal imaging camera (FOTRIC 220s; FOTRIC Thermal Intelligence, China) was employed to record changes in the solution's temperature. To assess the photothermal stability of MCM, temperature variations were also observed over five NIR on/off cycles.

### Spread Plate Method

To assess the anti‐biofilm effects of MCM, six experimental groups were established: control (treated with PBS), 400 µg ml^−1^ CM, 400 µg ml^−1^ MCM, NIR, 400 µg ml^−1^ CM + NIR, and 400 µg ml^−1^ MCM + NIR. Bacterial colony quantification was performed employing the spread plate method. The biofilms, which had undergone different treatments, were suspended in PBS, and a set of sequential tenfold dilutions was prepared. Afterward, 100 µL of the PBS dilution was uniformly distributed onto blood agar plates. Following an cultivation period of 24 h at 37 °C, the bacterial colonies were enumerated.

### Crystal Violet Staining

Following the removal of the medium, crystal violet solution was used to stain the biofilms. Each group underwent thorough washing and drying. Following this, the biofilms were dissolved and the absorbance of the resulting eluate at a wavelength of 590 nm was measured using a microplate reader (BioTek Instruments, Winooski, VT, USA).

### Scanning Electron Microscopy

The biofilm, cultured on titanium sheets and subjected to various treatments (control, CM, MCM, NIR, CM+NIR, MCM+NIR), was fixed with 2.5% glutaraldehyde at 4 °C overnight. Subsequent dehydration involved using graded ethanol (30%, 50%, 70%, 80%, 95%, and 100%) for 10 min at each ethanol concentration. Following lyophilization and gold spraying, SEM was employed for biofilm observation.

### Bacterial Live/Dead Staining

To visualize the biofilm's 3D structure, it was cultured on discs in confocal dishes. Following various treatments, the biofilm was thoroughly washed and then stained with the LIVE/DEAD BacLight Bacterial Viability Kit, as per the manufacturer's instructions, while protected from light for an hour. Subsequently, the biofilm's structure was examined using a CLSM (ZEISS LSM 710, Germany). Biomass analysis was carried out using the COMSTAT plugin within the ImageJ software.

### RNA Sequencing

To conduct RNA‐seq analysis, total RNA was extracted from both the control and MCM groups (at a concentration of 400 µg mL^−1^) using TRIzol reagent. Subsequently, gene profiling and comprehensive data analysis were carried out by Oebiotech Co. Ltd (Shanghai, China). Data analysis encompassed correlation assessment, the analysis of differentially expressed genes (DEGs), Gene Ontology (GO) analysis, and KEGG analysis, all of which were performed using the Oebiotech cloud platform (https://cloud.oebiotech.cn/task/).

### Immunofluorescence Staining of Biofilm by Hypoxyprobe

*S. aureus* biofilm cultured on glass slides was pretreated with nanoparticles (100 µL) for 8 h. Subsequently, the extra nanoparticles were removed and pimonidazole hydrochloride was applied to the biofilm. After incubation of 90 min, specific primary antibodies were then added to the biofilm. Next, fluorescent‐labeled secondary antibodies were introduced to the biofilm to bind to the primary antibodies, each with a 1‐h incubation. All slides were observed under a fluorescence microscope (Olympus IX81).

### Enhanced Cuproptosis‐Like Death Induced by MCM

According to the manufacturer's instructions, the activity of bacterial respiratory chain complexes I and II in the biofilms was measured using the Mitochondrial Respiratory Chain Complex Activity Detection Kit (Solarbio, Beijing, China). For the assessment of lipid peroxidation, the lipid peroxidation probe C11 BODIPY 581/591 (Invitrogen, USA) was added to the biofilms, and fluorescence was observed using laser scanning confocal microscopy after a 1‐h incubation. The concentration of the lipid peroxidation metabolite MDA was measured following the manufacturer's instructions using the MDA detection assay kit (Jiancheng, Nanjing, China).

### Confocal Laser Scanning Microscopy

The RAW264.7 cells, which had undergone different treatments, were washed three times with PBS for 3 min each time. They were then fixed with 4% paraformaldehyde for 15 min, followed by three additional PBS washes, each for 3 min. Permeabilization was achieved by incubating the cells with 0.5% Triton X‐100 at room temperature for 20 min, followed by another PBS wash. The blocking step was carried out by adding 5% BSA at room temperature for 30 min. After removing the blocking solution, an appropriate amount of either rabbit anti‐mouse CCR7 antibody (1:100) or rabbit anti‐mouse CD206 antibody (1:50, Abcam) was added, and the cells were incubated at 4 °C overnight. The following day, the primary antibodies were removed, and Alexa Fluor 488‐labeled goat anti‐rabbit antibody (1:500, Abcam) was added to bind to CCR7, along with Alexa Fluor 594‐labeled goat anti‐rabbit antibody (1:500, Abcam) to bind to CD206. After a 1‐h incubation, the cells were washed three times with PBS, and DAPI was added for light‐protected incubation for 5 min. CLSM was used for image acquisition.

### Phagocytic Capability of Macrophages

To assess the phagocytic capability of macrophages, Green Fluorescent protein‐labeled bacteria were co‐incubated with macrophages at a multiplicity of infection (MOI) of 10:1 for 2 h. After the removal of free‐floating bacteria and cell fixation with paraformaldehyde, rhodamine‐labeled phalloidin was added to stain the cells. Finally, the cells were observed using a fluorescence microscope.

### Flow Cytometry

The treated dendritic cells and RAW264.7 cells were collected in Eppendorf tubes, the culture medium was removed, and the cells were washed three times with PBS. For dendritic cells, Alexa Fluor 488‐labeled anti‐CD80 antibody and APC‐labeled anti‐CD86 antibody were added, and for RAW264.7 cells, APC‐labeled anti‐CD86 antibody and PE‐labeled anti‐CD206 antibody were added to the cells. Then they were chilled on ice for 30 min. Subsequently, the proportion of M1 and M2 macrophages was detected using a flow cytometer (Beckman Coulter, USA).

### Enzyme‐Linked Immunosorbent Assay

The concentrations of the pro‐inflammatory cytokine TNFα and the anti‐inflammatory cytokine IL‐10 were used to reflect the cell's inflammatory status. The supernatant of RAW264.7 cells after various treatments was collected. Subsequently, the concentrations of cytokines were measured using ELISA kits following the manufacturer's instructions.

### Osteogenesis and Angiogenesis Assays (In Vitro)

Genes related to osteogenic differentiation, *BMP2*, *OCN*, and *RUNX*, were assessed using RT‐qPCR to reflect the osteogenic differentiation of BMSCs. Cells cultured under different conditions for 14 days were collected, and RNA was extracted using the RNA Purification Kit (EZBionscience) following the manufacturer's instructions. RNA was reverse‐transcribed into cDNA using the Color Reverse Transcription Kit (EZBionscience). RT‐qPCR was performed with cDNA using 2 × Color SYBR Green qPCR Master Mix (EZBionscience) on the QuantStudio 7 Flex system (Life Technologies, Carlsbad, CA, United States). Sequences of all primers are provided in Table S1 (Supporting Information).

For the ALP assay, cells cultured for 7 and 14 days were subjected to ALP activity measurements using the Alkaline Phosphatase Assay Kit (ab83369, Abcam, Cambridge, UK) following the manufacturer's instructions. Protein content was quantitatively analyzed using the BCA protein assay kit (Solarbio, China). For the ARS experiment, BMSCs cultured for 14 days were fixed with paraformaldehyde and stained with Alizarin Red (pH 4.2) for 20 min. After washing the samples with PBS, they were observed and photographed using an optical microscope.

Tube formation assay was conducted to assess the angiogenesis capacity of MCM. Matrigel (BD, Franklin Lakes, NJ, USA) was used to coat 24‐well plates entirely. After 6 h incubation, the cells were observed with an optical microscope, and the number of nodes and junctions were analyzed.

### Implant‐Associated Infections Model Construction

All male BALB/c mice and SD rats were provided by the Animal Laboratory of Shanghai Sixth People's Hospital. All the animal procedures were performed under the protocols approved by Animal Welfare Ethics Committee of Shanghai Sixth People's Hospital (DWLL‐2023‐0527). Twenty male mice, aged 6–8 weeks, were randomly divided into four groups: control, CM, MCM, and MCM + NIR. Initially, titanium disks were incubated in a bacterial suspension for 48 h to form *S. aureus* biofilms on their surfaces. After creating an ≈1 cm incision on the backs of the mice, the titanium disks were implanted subcutaneously. On the second day, nanomaterials were introduced, and laser treatment (1 W cm^−2^, 10 min) was applied. Local infection status was documented through photography on days 1, 5, 7, and 10. Spread plate method and scanning electron microscopy were employed to measure the severity of local tissue infections and immunofluorescence staining were conducted for histological analysis by paraffin sections. The main organ's quantitative elemental analysis was conducted using ICP‐MS (Agilent 7800).

### Bacterial Infected Bone Defect Model Construction

Twenty male rats, aged 9 weeks, were randomly divided into four groups: control, CM, MCM, and MCM+NIR. Using a drill, a 0.6 mm diameter defect was created in the femoral condyle of the rats, and 10^7^ CFU of *S. aureus* was introduced to establish a bone infection model. The bone defects were then covered. Nanomaterials were injected and laser treatment (1 W cm^−2^, 10 min) was applied afterward on the second day. Regenerated bone was observed by µCT images. Giemsa, Masson and immunofluorescence staining were employed for histological analysis by paraffin sections.

### Bacterial Colony Counting

Experimental analysis was performed on 1 g of tissue surrounding the infection site. The isolated tissues were ground into a homogenate and subsequently diluted in PBS. Bacterial colony counting was then conducted following the spread plate method outlined in the in vitro experiments.

### Statistical Analysis

The in vitro experiments were repeated on a minimum of three occasions, while the in vivo experiments were conducted at least six times. All data were presented as the mean ± standard deviation and were subjected to statistical analysis using GraphPad Prism software (version 9.0) or Origin (Version 2019b). Statistical significance was determined through Student's t‐tests and one‐way analysis of variance (ANOVA) tests, with the threshold for significance set at P <0.05. The levels of significance were indicated as follows: ^*^ *p* <0.05, ^**^ *p* <0.01, ^***^ *p* <0.001, ^****^ *p* <0.0001, and ns indicating no statistical significance.

## Conflict of Interest

The authors declare no conflict of interest.

## Supporting information

Supporting Information

## Data Availability

The data that support the findings of this study are available from the corresponding author upon reasonable request.
