# Supplementary material for: Enhanced Bacterial Cuproptosis‐Like Death via Reversal of Hypoxia Microenvironment for Biofilm Infection Treatment
Source: Adv Sci (Weinh). 2024 Mar 13;11(19):2308850. doi: 10.1002/advs.202308850 (PMC11109650; doi:10.1002/advs.202308850)
Supplement: Supplementary file 1 — Supporting Information [file ADVS-11-2308850-s001.pdf]

ADVANCED SCIENCE
Open Access

Supporting Information

for *Adv. Sci.*, DOI 10.1002/advs.202308850

Enhanced Bacterial Cuproptosis-Like Death via Reversal of Hypoxia Microenvironment for Biofilm Infection Treatment

Zhiyuan Luo, Renjie Lu, Tingwang Shi, Zesong Ruan, Wenbo Wang, Zhao Guo, Zeming Zhan, Yihong Ma, Xiaofeng Lian, Cheng Ding* and Yunfeng Chen**

Supporting Information

Enhanced Bacterial Cuproptosis-like Death via Reversal of Hypoxia Microenvironment for Biofilm Infection Treatment

Zhiyuan Luo[1], Renjie Lu[1], Tingwang Shi, Zesong Ruan, Wenbo Wang, Zhao Guo, Zeming Zhan, Yihong Ma, Xiaofeng Lian, Cheng Ding*, Yunfeng Chen**

Materials

Copper acetate monohydrate, 1,3,5-Benzenetricarboxylic acid (H_3BTC) and $KMnO_4$ were purchased from Macklin Reagent Co. (China). BODIPY 581/591 C11 probe were obtained from Thermo Fisher Scientific (Waltham, MA, USA). RNAprep Pure Cell/Bacteria Kit, the Color Reverse Transcription Kit and 2 × Color SYBR Green qPCR Master Mix were obtained from EZBionscience. Alexa Fluor 488-labeled anti-CD80 antibody, APC-labeled anti-CD86 antibody and PE-labeled anti-CD206 antibody were obtained from BioLegend (USA). ELISA kits for TNF-α and IL-10 were obtained from Multi Science Biotech Co. (China), while Rhodamine-labeled phalloidin was obtained from Yeasen (Shanghai, China).

Bacterial strains and biofilm culture

Staphylococcus aureus (*S. aureus*, ATCC 43300) was utilized in this study. Planktonic bacteria were cultured overnight in tryptic soy broth (TSB) at 37°C and 250 rpm. For biofilm cultivation, a bacterial suspension of 10^6 CFU was added to titanium discs placed in 24-well plates and incubated at 37°C overnight. Subsequently, the bacterial suspension was removed for further experiments.

In vitro cytotoxicity

HUVEC, RAW264.7, and BMSC cells were cultured in dulbecco's modified eagle medium supplemented with 10% fetal bovine serum, 100 U mL^{-1} penicillin, and 100 μg

mL^{-1} streptomycin. The culture conditions included 5% CO_2 at 37°C. For the CCK8 assay, cells were seeded into a 96-well plate at a density of 8000 cells per well. After overnight incubation, different concentrations of MCM (0, 50, 100, 200, 400, 800 μg mL^{-1}) were added. Following 24 hours of incubation, the culture medium was removed, and CCK-8 solution was added. After 1.5 hours of incubation, the absorbance at 450nm was measured using a microplate reader (Epoch BioTEK, USA).

Supplementary Tables and Figures

Table S1. Primers sequences used in PCR experiment.[1]

Gene	Upper primer sequence (5' to 3')	Lower primer sequence (5' to 3')
GAPDH	ACAGTTGCCATGTAGACC	TTTTTGGTTGAGCACAGG
BMP2	TCCACCATGAAGAATCTTTG	TAATTCGGTGATGGAAACTG
OCN	TTCTTTCCTCTTCCCCTTG	CCTCTTCTGGAGTTTATTTGG
Runx2	AAGCTTGATGACTCTAAACC	TCTGTAATCTGACTCTGTCC

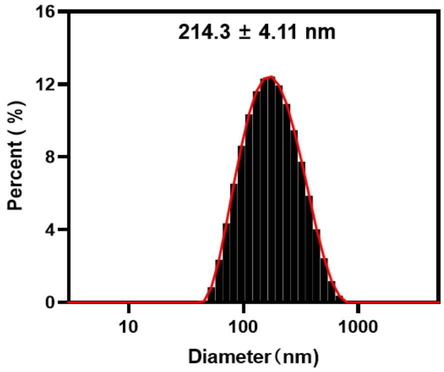

Figure S1. Particle size distributions of CM.

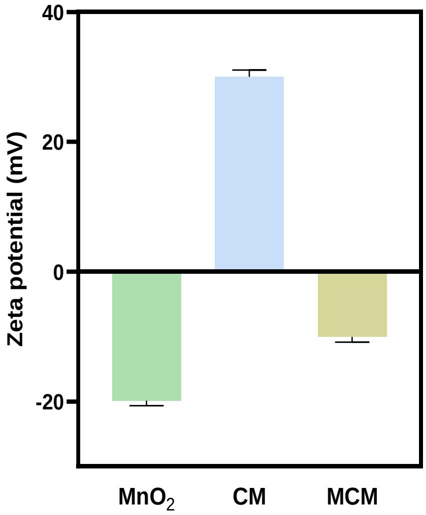

Figure S2. Zeta potential of CM and MCM solution.

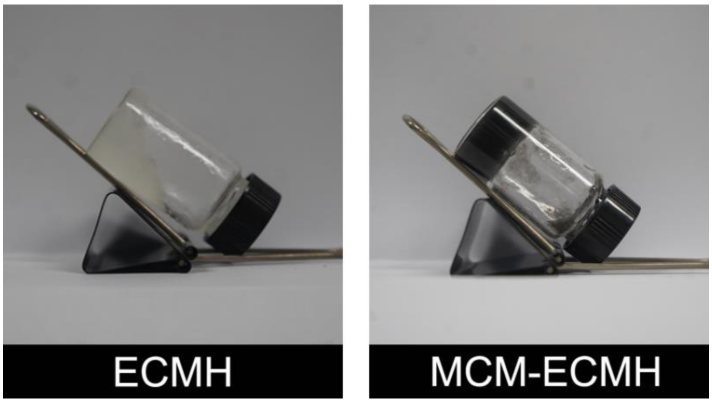

Figure S3. Representative photographs of ECMH and MCM-ECMH after placed for 1 h.

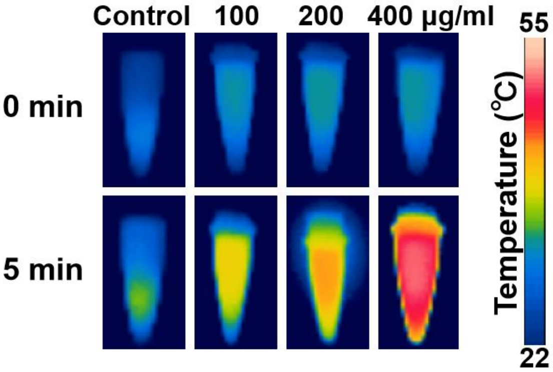

Figure S4. Photothermal images of MCM nanoparticles under 808 nm laser irradiation (1 W cm^{-2}, 5 min).

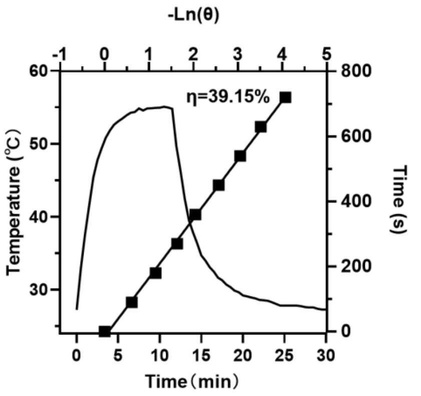

Figure S5. Photothermal conversion efficiency of MCM under 808 nm laser irradiation.

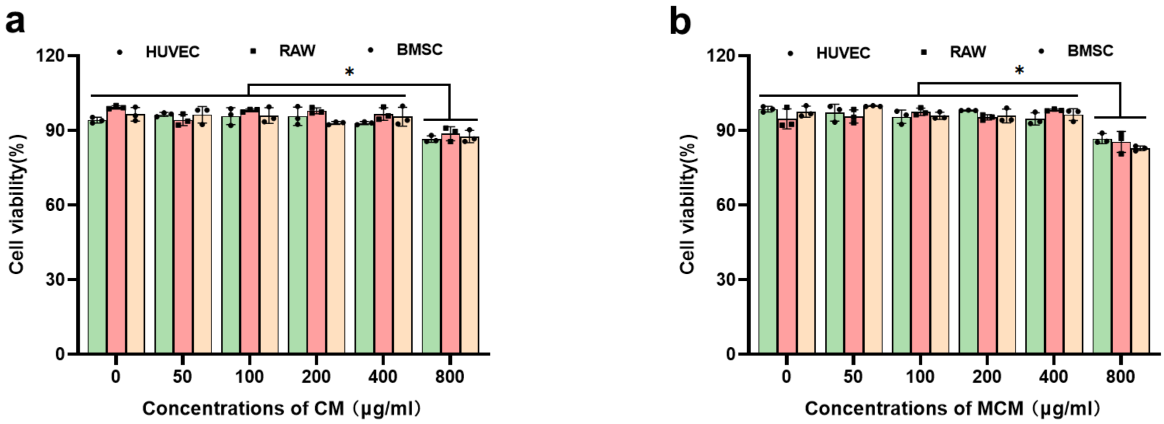

Figure S6. Cell viability of HUVEC, RAW264.7 and BMSC cells accessed by CCK8 assay after cultured with different concentrations of (a) CM and (b) MCM.

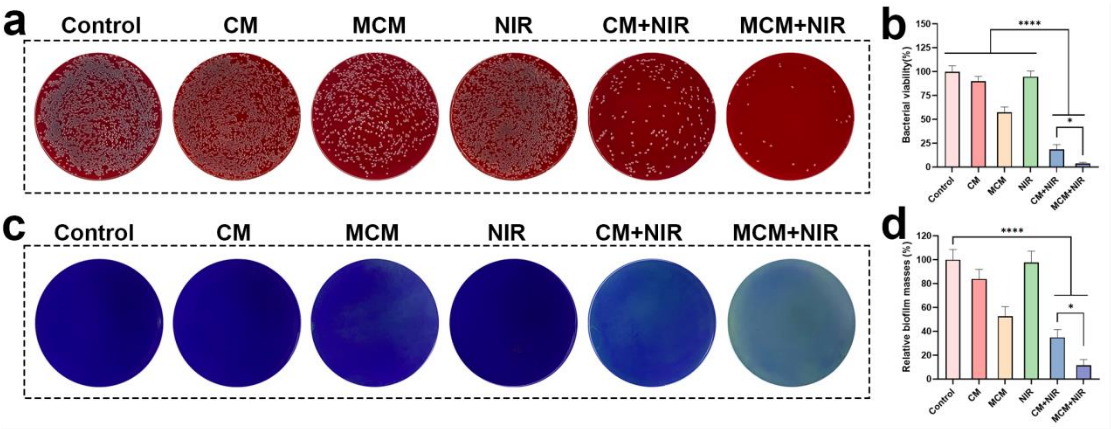

Figure S7. (a) Representative photographs depicting bacterial colonies within *E. coli* biofilms using SPM. (b) Quantitative evaluation of bacterial viability via SPM. (c) Digital images of the *E. coli* biofilms stained with crystal violet. (d) Biomass of *E. coli* biofilm. Data are presented as mean ± s.d, n = 3, * $p < 0.05$ and ****$p < 0.0001$.

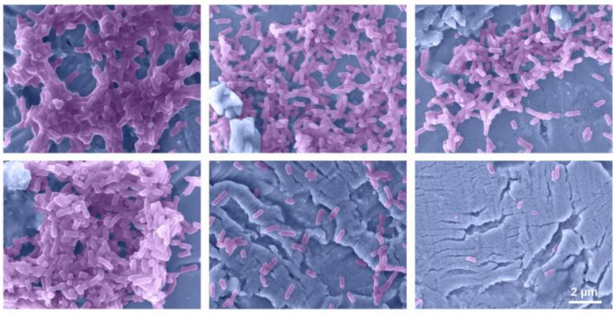

Figure S8. SEM images of *E. coli* biofilms. Scale bar, 2 μm.

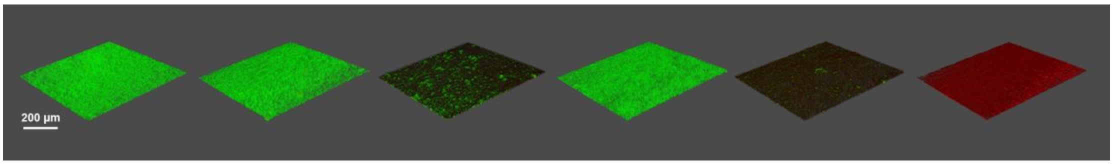

Figure S9. 3D reconstructions images presenting *E. coli* biofilm stained with SYTO 9/PI. Scale bar, 200 μm.

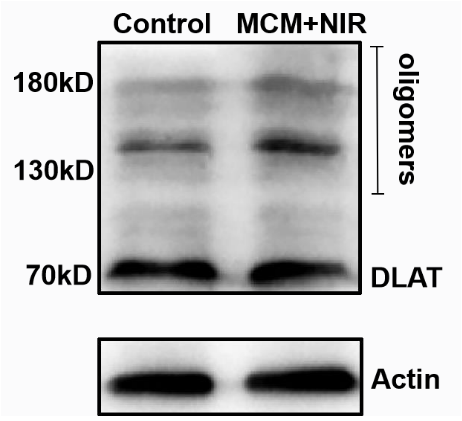

Figure S10. Bacterial proteins analyzed for DLAT oligomerization.

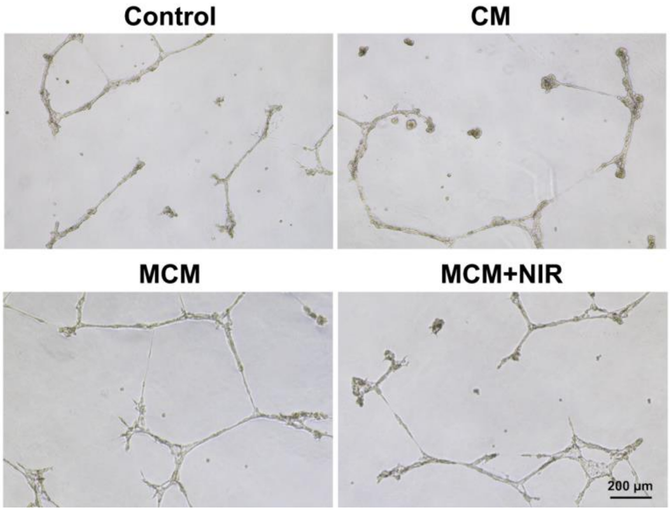

Figure S11. Representative images of tubular formation of HUVECs after 6 hours incubation. Scale bar, 200 μm.

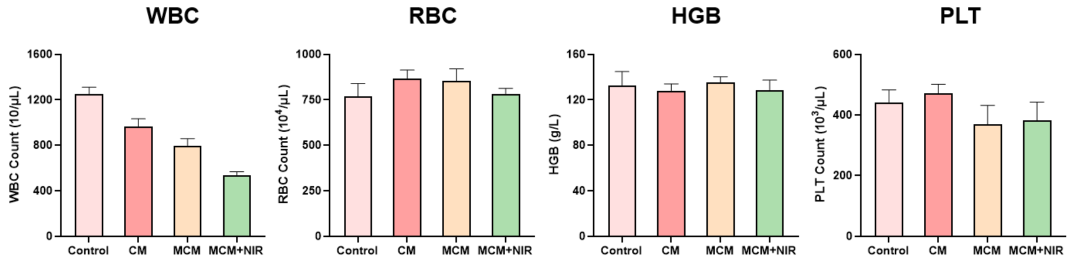

Figure S12. (a) WBC, (b) RBC, (c) HGB, and (d) PLT cell counts in the peripheral blood of mice in various groups.

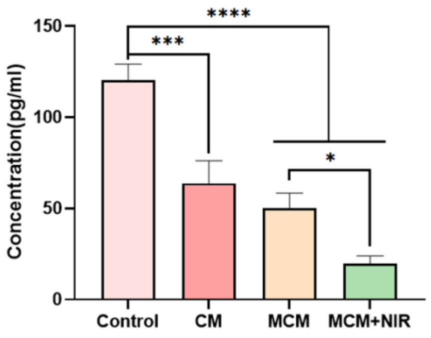

Figure S13. ELISA assay of IL-10 after different treatments.

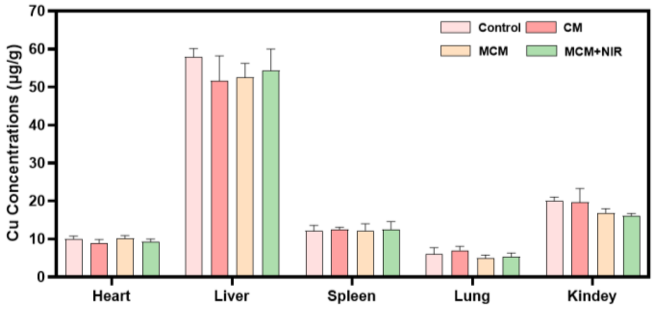

Figure S14. In vivo copper biodistribution measured by ICP-MS.

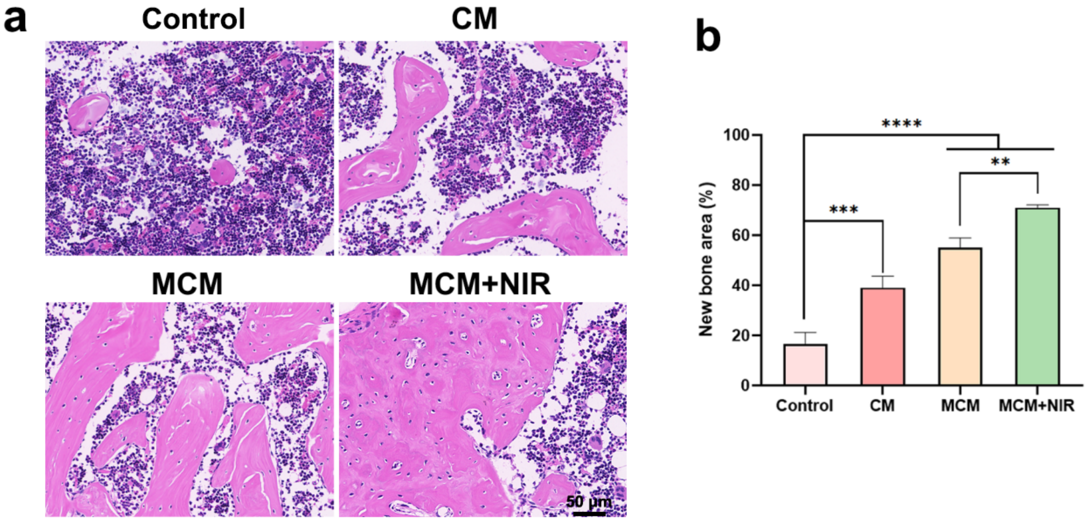

Figure S15. (a) H&E staining images of the infected tissue and (b) quantitative analyses of H&E staining. Scale bar, 50 μm.

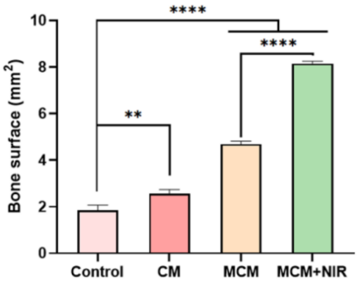

Figure S16. Quantitative analyses of bone surface.

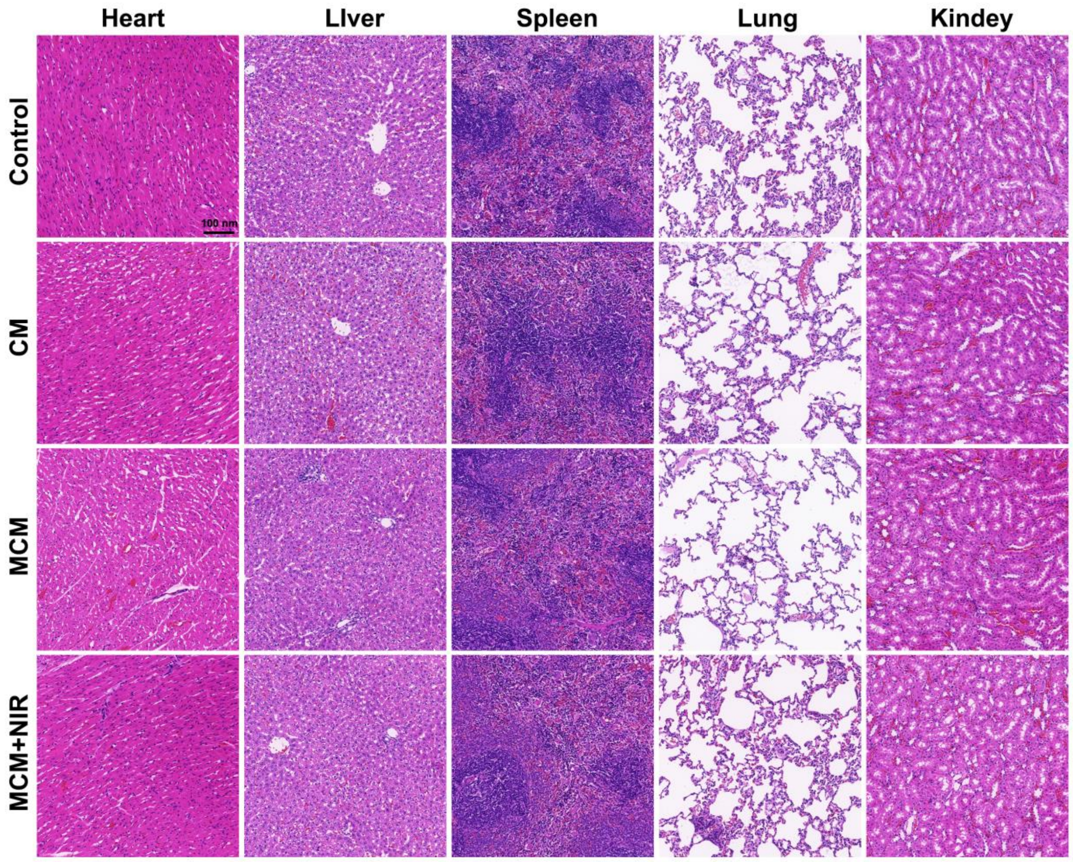

Figure S17. H&E staining images of major organs of the rats (heart, liver, spleen, lung and kidney). Scale bar, 100 μm.

Supplementary References

[1] a)Y. Huang, X. Zhai, T. Ma, M. Zhang, H. Yang, S. Zhang, J. Wang, W. Liu, X. Jin, W. W. Lu, X. Zhao, W. Hou, T. Sun, J. Shen, H. Pan, Y. Du, C. H. Yan, *Advanced Materials* **2023**, 35, e2300313; b)O. R. Mahon, D. C. Browe, T. Gonzalez-Fernandez, P. Pitacco, I. T. Whelan, S. Von Euw, C. Hobbs, V. Nicolosi, K. T. Cunningham, K. H. G. Mills, D. J. Kelly, A. Dunne, *Biomaterials* **2020**, 239, 139833.
